# Radiographic Outcomes of Open Reduction and Kirschner-Wire Fixation Versus Screw or Plate Fixation for Lisfranc Injuries: A Propensity Score-Weighted Comparative Study

**DOI:** 10.3390/jcm15187248

**Published:** 2026-09-18

**Authors:** Woo-sung Choi, Jae-hyeon Seo, Youngrak Choi

**Affiliations:** 1 Department of Orthopedic Surgery, Asan Medical Center, Seoul 05505, Republic of Korea; wooseong96@naver.com; 2 Department of Orthopedic Surgery, Suwon Nanoori Hospital, Suwon 16503, Republic of Korea; jhseo112@gmail.com

**Keywords:** Lisfranc injury, K-wire fixation, propensity score overlap weighting, radiographic outcome, posttraumatic osteoarthritis

## Abstract

**Background/Objectives:** Accurate anatomic reduction is a major determinant of outcomes after Lisfranc injury, but the optimal fixation method remains controversial. Although screws/plates are commonly used, Kirschner-wire (K-wire) fixation may provide adequate stability after precise open reduction. **Methods:** We retrospectively reviewed 62 patients who underwent surgery for acute unstable Lisfranc injuries at a single institution between 2002 and 2024. Fifty patients underwent open reduction and K-wire fixation, and 12 underwent screw/plate fixation. Propensity score overlap weighting was performed using age, sex, body mass index, time from injury to surgery, and injury mechanism. The primary outcome was final follow-up medial cuneiform-second metatarsal (C1-M2) gap; Post-traumatic osteoarthritic change was the secondary outcome. **Results:** After overlap weighting, baseline covariates were well balanced. The K-wire group had a significantly smaller final C1-M2 gap than the screw/plate group (1.919 ± 0.115 mm vs. 2.536 ± 0.279 mm, *p* = 0.046; weighted mean difference, 0.617 mm; 95% confidence interval, 0.013–1.220). Post-traumatic osteoarthritic changes were less frequent in the K-wire group (29.6% vs. 57.7%), although the difference was not significant (*p* = 0.091). **Conclusions:** When accurate open reduction was achieved, K-wire fixation provided radiographic outcomes comparable to and, regarding the C1-M2 gap, potentially more favorable than screw/plate fixation. Despite reduced construct rigidity, multiple K-wire fixations appeared to provide sufficient stability to maintain anatomic reduction and may reduce subtle reduction loss during screw/plate insertion. These findings suggest that K-wire fixation is a reasonable, less articular-invasive fixation option for acute unstable Lisfranc injuries.

## 1. Introduction

Lisfranc injuries comprise a spectrum of tarsometatarsal joint injuries ranging from subtle ligamentous instability to fracture dislocation. Subtle Lisfranc injury has been described as 1–5 mm of midfoot diastasis, but even these injuries can result in chronic pain, midfoot deformity, post-traumatic osteoarthritis, and functional disability if missed or inadequately treated [1,2,3,4,5,6].

For unstable or displaced Lisfranc injuries, operative treatment is generally recommended to restore the anatomic alignment of the medial and central columns [4,5,7]. Reduction quality has been reported to be closely associated with long-term clinical and radiographic outcomes. Favorable results are more common after anatomic reduction, whereas malreduction is associated with unsatisfactory outcomes and post-traumatic arthritis [2,8,9,10].

Several fixation strategies have been described, including transarticular screw fixation, dorsal bridge plating, percutaneous fixation, flexible fixation, and primary arthrodesis [7,11,12,13,14,15,16,17,18,19]. However, the optimal fixation method remains controversial. Although rigid implants are intended to maintain reduction, they have limitations. Transarticular screws may violate the articular surface or break, and often require secondary removal. Dorsal bridge plates avoid direct transarticular fixation but may cause implant irritation and usually require removal. Primary arthrodesis has also gained attention because of the reported high rates of post-traumatic osteoarthritis and secondary fusion after fixation in some series [7,11,12,13,14,15,16].

Compared with screws or plates, K-wire fixation has traditionally been regarded as a less rigid construct or provisional fixation, particularly for high-energy injuries or severe soft-tissue damage [20,21]. Biomechanical work has suggested that K-wire-only construct may be less rigid than screw-based fixation [22]. Nevertheless, the Lisfranc joint has inherent bony and ligamentous stability, including the recessed second metatarsal base and the surrounding dorsal, interosseous, and plantar ligamentous complex [23,24]. Therefore, if accurate open reduction restores the native tarsometatarsal architecture, multiple K-wires may provide sufficient stability during soft tissue healing while limiting implant bulk and articular injury.

We hypothesized that, when accurate open reduction and stable fixation are achieved, K-wire fixation would not result in inferior radiographic outcomes compared with screw/plate fixation. Accordingly, the main question addressed in this study was whether open reduction with multiple K-wire fixation could maintain radiographic reduction comparably to screw or plate fixation in patients with acute unstable Lisfranc injuries. Therefore, this study compared the radiographic outcomes of K-wire and screw/plate fixation for acute unstable Lisfranc injuries using propensity score overlap weighting to reduce baseline imbalance between treatment groups.

## 2. Materials and Methods

### 2.1. Study Design and Patient Selection

This retrospective comparative study included patients who underwent operative treatment for acute unstable Lisfranc injury at a single institution between 2002 and 2024.

Patients were eligible if they had an acute unstable Lisfranc injury involving the medial and/or central column and underwent open reduction with K-wire fixation alone or screw/plate fixation. Patients with chronic or neglected injuries were excluded. The final cohort included patients with a minimum 2-year follow-up and adequate postoperative and follow-up weight-bearing radiographs for assessment.

A total of 62 patients met the study criteria; 50 underwent K-wire fixation and 12 underwent screw or plate fixation. Demographic and injury-related variables, including age, sex, body mass index (BMI), time from injury to surgery, and injury mechanism (high- or low-energy trauma), were reviewed.

### 2.2. Operative Technique

All procedures were performed under general or spinal anesthesia. Instability of the involved tarsometatarsal joints was assessed by intraoperative stress examination under fluoroscopy and direct exploration. Open reduction was performed through dorsal longitudinal incisions centered over the involved joints. Anatomic reduction was achieved manually and confirmed primarily by direct visualization, with fluoroscopy used to assess overall alignment and implant position. The choice between K-wire and screw/plate fixation was not based on a predefined severity-based algorithm or intentional selection according to injury severity, injury location, or intraoperative instability. Instead, the fixation method largely reflected implant availability and changes in institutional practice during the study period.

**Figure 1:**
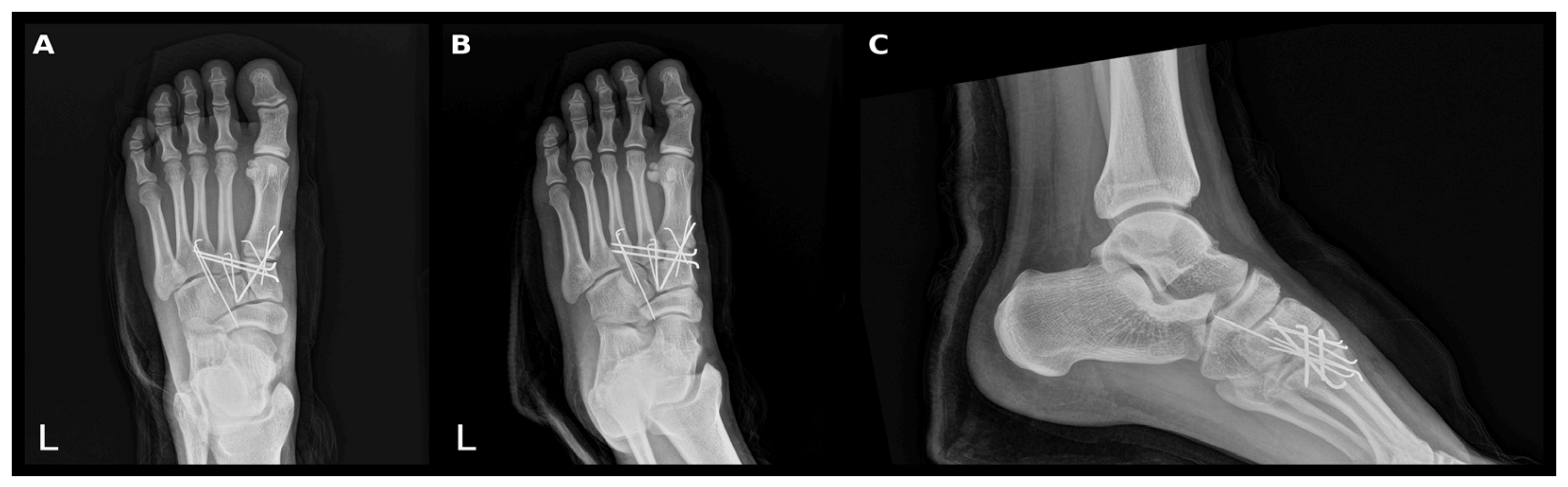
Operative technique of K-wire fixation for acute unstable Lisfranc injury. Postoperative anteroposterior (**A**), oblique (**B**), and lateral (**C**) radiographs illustrate anatomic reduction in the tarsometatarsal complex and stabilization with multiple K-wires inserted in crossed or divergent trajectories across the involved joints.

In the K-wire fixation group, the involved joints were anatomically reduced and stabilized using multiple K-wires. No. 3 K-wires, corresponding to a diameter of 1.6 mm or 0.062 inches, were generally used. Depending on the involved joint and fracture configuration, two or three K-wires were inserted across each unstable joint to obtain stable fixation. Fixation was generally performed sequentially from the medial to lateral column, with crossed or divergent wires selected according to the involved joint and fracture configuration. Because single-wire fixation may be mechanically weaker than screw fixation, multiple K-wires were used to increase construct stability when necessary [22]. A representative radiographic illustration of the K-wire fixation technique is provided in Figure 1, demonstrating postoperative reduction and the typical wire configuration.

In the screw/plate fixation group, fixation was achieved using transarticular screws, dorsal bridge plates, or both, depending on implant availability, contemporary practice, and surgeon preference. Transarticular screws were inserted across the unstable tarsometatarsal joints, whereas dorsal bridge plates were applied dorsally to maintain reduction and stability of the involved joints. After definitive fixation, reduction maintenance and implant position were reassessed using direct visualization and fluoroscopy.

The general postoperative protocol was similar between the two fixation groups. Patients were initially immobilized and kept non-weight-bearing. Weight-bearing was generally initiated at approximately 6 weeks postoperatively using a medial arch-support insole, regardless of whether K-wire or screw/plate fixation had been performed. Use of the medial arch-support insole was recommended for at least 6 months after surgery. Internal fixation devices were generally retained for at least 6 months in both groups. Elective implant removal, when requested by the patient or clinically indicated, was usually considered after 1 year postoperatively. Although postoperative immobilization, weight-bearing progression, and implant removal timing were adjusted slightly according to postoperative symptoms, fixation stability, soft-tissue condition, and surgeon judgment, most patients followed this general protocol.

### 2.3. Radiographic Assessment

Radiographic assessment focused on maintenance of reduction and development of post-traumatic osteoarthritic change. The primary outcome was the C1-M2 gap, defined as the distance between the medial cuneiform and the base of the second metatarsal, measured on final follow-up weight-bearing anteroposterior radiographs. Because non-weight-bearing radiographs may underestimate subtle Lisfranc instability, weight-bearing radiographs were used whenever available for follow-up assessment [25].

Malreduction or reduction loss was defined as a final follow-up C1-M2 gap > 2 mm on weight-bearing anteroposterior radiographs [1,25]. Post-traumatic osteoarthritic change was evaluated on final follow-up radiographs using the Kellgren–Lawrence grading system [26].

### 2.4. Statistical Analysis

To minimize confounding between the two surgical groups, propensity score (PS) weighting was used [27,28,29]. Propensity scores were estimated using a logistic regression model, including age, sex, BMI, trauma mechanism, and time to surgery.

Because the K-wire group was substantially larger than the screw/plate group, conventional propensity score matching would have discarded many K-wire cases and reduced statistical power. Therefore, overlap weighting was selected instead of 1:1 matching. This approach retains all eligible patients while assigning greater weight to those whose baseline characteristics make either treatment option plausible, creating a weighted population with better baseline covariate balance.

Specifically, overlap weighting (average treatment effect in the overlap population) was applied to achieve covariate balance. Patients were weighted according to the probability of receiving the opposite treatment (1—PS for the treatment group and PS for the control group), emphasizing those who could reasonably have received either fixation method and enabling a more balanced comparison.

Following application of overlap weighting, baseline covariate balance was assessed using standardized mean differences (SMDs), with an SMD of <0.1 indicating negligible imbalance. Radiographic outcomes were compared using design-based *t*-tests and Rao–Scott adjusted chi-square tests within a survey-weighted framework to account for the weighted data structure. Statistical analyses were performed using R version 4.3.2 with the WeightIt, cobalt, and survey packages. Statistical significance was set at *p* < 0.05.

## 3. Results

### 3.1. Baseline Characteristics

Before weighting, the K-wire and screw/plate fixation groups comprised 50 and 12 patients, respectively. Baseline characteristics are summarized in Table 1. Although conventional *p*-values showed no significant between-group differences, SMDs indicated meaningful baseline imbalance, particularly for sex (0.450), time from injury to surgery (0.433), and BMI (0.298).

**Figure 2:**
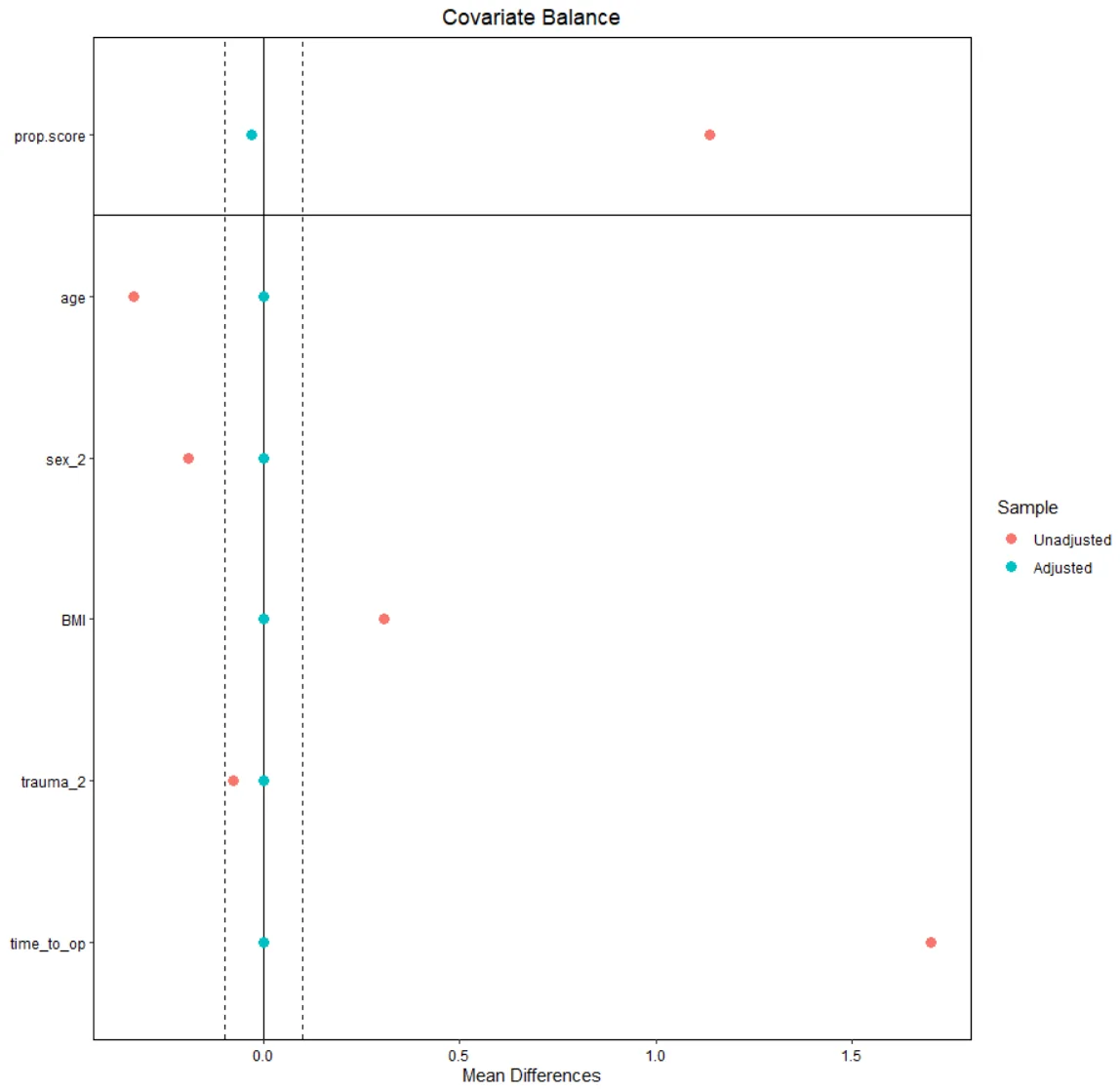
Covariate balance before and after overlap weighting. The Love plot shows absolute standardized mean differences before and after overlap weighting. Dashed vertical reference lines indicate conventional thresholds for covariate balance; adjusted values were centered near zero after weighting.

After overlap weighting, covariate balance improved substantially. In the weighted population, all included baseline covariates had adjusted SMDs below the conventional threshold of 0.1 (Figure 2).

### 3.2. Radiographic Outcomes

Radiographic outcomes before and after overlap weighting are summarized in Table 2. Raw (unweighted) outcomes are presented for descriptive transparency; however, primary inferences were based on the weighted comparison because baseline imbalance existed before weighting. After overlap weighting, the K-wire fixation group showed a significantly smaller final C1-M2 gap than the screw/plate fixation group (1.919 ± 0.115 mm vs. 2.536 ± 0.279 mm, *p* = 0.046), with a weighted mean difference of 0.617 mm (95% confidence interval [CI], 0.013–1.220).

Post-traumatic osteoarthritic changes were also less frequent in the K-wire group than in the screw/plate fixation group (29.6% vs. 57.7%) after overlap weighting, although the difference was not statistically significant (*p* = 0.091).

## 4. Discussion

The principal finding of this study was that K-wire fixation achieved radiographic outcomes comparable to those of screw or plate fixation after adjustment for baseline differences using propensity score overlap weighting. Contrary to concerns that a less rigid construct might increase reduction loss, K-wire fixation was not associated with a larger final C1-M2 gap. Instead, the weighted analysis demonstrated a smaller final C1-M2 gap in the K-wire group.

These findings support the concept that accurate anatomic reduction may be more important than fixation rigidity alone in determining radiographic outcomes after Lisfranc injury. This concept is consistent with prior studies showing that reduction quality is strongly associated with long-term outcomes, whereas malreduction is associated with persistent pain and post-traumatic arthritis [2,6,8,9,10].

Rigid fixation has traditionally been used to maintain reduction, and screw fixation, dorsal bridge plating, and primary arthrodesis remain important treatment options [7,11,12,13,14,15,16,17]. However, rigid constructs may be associated with implant irritation, articular cartilage violation, hardware failure, and the need for secondary implant removal. In addition, the relative benefits of open reduction and internal fixation versus primary arthrodesis remain debated, particularly regarding post-traumatic osteoarthritis and secondary fusion [7,11,12,13,14,15,16].

K-wire fixation may offer several potential advantages for acute unstable Lisfranc injuries when accurate open reduction has been achieved. K-wires are technically simple, create minimal implant bulk, and are easily removed. They may also reduce articular surface injury and hardware-related irritation associated with larger transarticular screws or plates. From a technical perspective, the fixation step itself can influence the final reduction. In our surgical experience, screw insertion or plate application after provisional reduction may occasionally disturb a precisely reduced tarsometatarsal relationship, even with cannulated screw techniques. Because even minimal residual displacement may be clinically relevant in the Lisfranc joint, fixation that maintains reduction during implantation may be advantageous. Multiple K-wires can be inserted with less manipulation of the reduced joint relationship, helping preserve precise alignment after open reduction.

**Figure 3:**
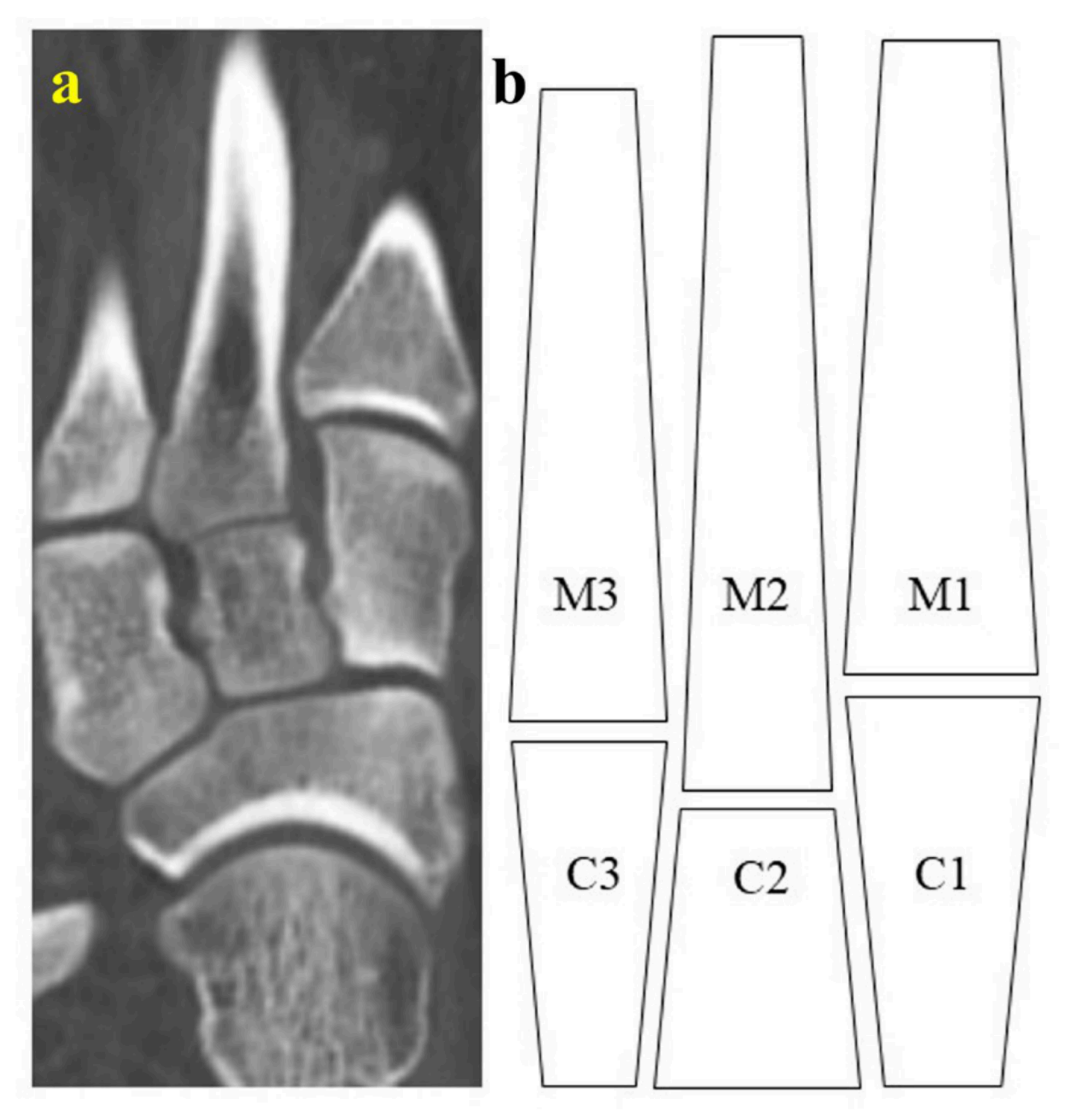
Coronal-plane bony structural stability of the Lisfranc joint. (**a**) Coronal computed tomography image and (**b**) schematic illustration showing the recessed, dovetail-shaped base of the second metatarsal between the cuneiforms, which limits lateral and distal translation of the adjacent metatarsals. C1, medial cuneiform; C2, intermediate cuneiform; C3, lateral cuneiform; M1, first metatarsal; M2, second metatarsal; M3, third metatarsal.

The intrinsic architecture of the Lisfranc joint may help explain why K-wire fixation can maintain reduction after accurate open reduction. In the coronal plane, the base of the second metatarsal is recessed between the medial and lateral cuneiforms in a dovetail-like configuration. This bony interlocking structure limits lateral and distal translation of adjacent metatarsal bases and provides inherent osseous stability once the tarsometatarsal relationship is anatomically restored (Figure 3) [23,24].

**Figure 4:**
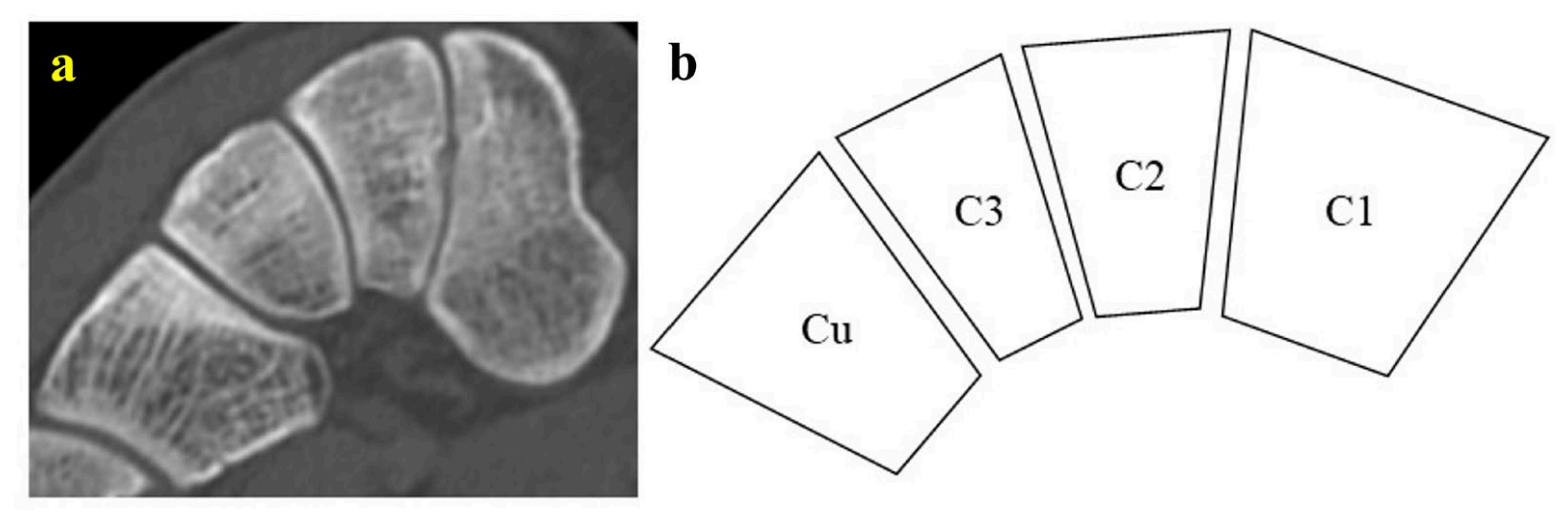
Axial-plane bony structural stability of the Lisfranc joint. (**a**) Axial computed tomography image and (**b**) schematic illustration showing the Roman arch-like configuration of the cuneiforms and cuboid. The broader dorsal than plantar surfaces contribute to intrinsic transverse stability of the tarsometatarsal complex. C1, medial cuneiform; C2, intermediate cuneiform; C3, lateral cuneiform; Cu, cuboid.

In the axial cross-sectional views, the cuneiforms and cuboid form an arch-like configuration, with broader dorsal than plantar surfaces. This transverse arch-like morphology contributes to transverse midfoot stability by resisting plantar migration and collapse of the cuneiforms and metatarsal bases after reduction (Figure 4). Together with the dorsal, interosseous, and plantar ligaments and the joint capsules, this osseous architecture provides a biologic rationale for the use of K-wire fixation during ligamentous and capsular healing [23,24].

This concept does not negate the mechanical value of rigid fixation. Rather, it suggests that once anatomic osseous alignment has been restored, the fixation construct may primarily need to maintain this relationship until soft-tissue healing occurs.

The present study should not be interpreted as evidence that K-wire fixation is universally superior to screw or plate fixation. Rather, our findings suggest that K-wire fixation may be sufficient when precise open reduction and stable fixation are obtained. The recent multicenter study by Adachi et al. directly compared K-wire fixation with screw fixation for Lisfranc injuries and reported comparable clinical outcomes between the two methods, but also found a higher malunion rate in the K-wire group [30]. This finding highlights a potential limitation of K-wire fixation when accurate reduction or adequate fixation stability is not achieved. By contrast, in the present study, reduction was achieved through open reduction and confirmed primarily by direct visualization, and the involved joints were stabilized using multiple K-wires. Under these conditions, the K-wire group showed no greater final C1–M2 gap widening than the screw/plate group after overlap weighting. Therefore, the differing radiographic findings should be interpreted in light of differences in reduction method, fixation technique, and radiographic outcome assessment.

This study has certain limitations. First, although injury mechanism was included as an available indicator of injury severity, detailed preoperative radiographic severity parameters, including preoperative C1–M2 gap, degree of displacement and fracture comminution, were not included in the propensity score model. This was primarily because standardized preoperative weight-bearing radiographs were not consistently available, as many patients could not tolerate weight-bearing imaging in the acute post-injury setting because of pain, swelling, or fracture-dislocation. Therefore, residual confounding related to initial injury severity and fixation selection cannot be completely excluded. Second, the small screw/plate group and the resulting relatively small weighted effective sample size limited statistical power and precision, particularly for categorical outcomes such as post-traumatic osteoarthritis. Therefore, the weighted estimates should be interpreted cautiously, and the generalizability of these findings to broader Lisfranc injury populations may be limited. Finally, this study focused on radiographic outcomes and did not include standardized clinical outcome measures such as AOFAS midfoot scores, VAS pain scores, return to activity, or patient satisfaction. Therefore, we cannot determine whether the observed radiographic differences translated into clinically meaningful benefits. Longer follow-up and future studies with larger cohorts combining radiographic assessment with patient-reported clinical outcomes are needed because post-traumatic osteoarthritis after Lisfranc injury may progress over several years [31].

## 5. Conclusions

In acute unstable Lisfranc injuries, K-wire fixation after accurate open reduction provided satisfactory radiographic outcomes without inferior reduction maintenance compared with screw or plate fixation. The smaller final C1-M2 gap in the K-wire group suggests that fixation rigidity alone may not determine radiographic outcomes. K-wire fixation may therefore be a reasonable, less articular-injurious alternative when precise open reduction and stable fixation are achieved.

## Figures and Tables

**Table 1:** Baseline characteristics before overlap weighting.

Variable	Level	K-Wire Fixation (*n* = 50)	Screw/ Plate Fixation (*n* = 12)	*p*-Value	SMD
*N*		50	12		
sex (%)	male	32 (64.0)	10 (83.3)	0.346	0.450
	female	18 (36.0)	2 (16.7)		
age (mean (SD))		39.24 (17.46)	34.67 (12.15)	0.395	0.304
BMI (mean (SD))		24.46 (4.29)	25.91 (5.32)	0.322	0.298
time_to_op (median [IQR])		7.00 [5.00, 10.00]	6.00 [3.75, 10.25]	0.907	0.433
trauma (%)	High	17 (34.0)	5 (41.7)	0.871	0.159
	Low	33 (66.0)	7 (58.3)		

Values are presented as number (%), mean (SD), or median [IQR], as appropriate. BMI, body mass index; IQR, interquartile range; SMD, standardized mean difference.

**Table 2:** Radiographic outcomes before and after overlap weighting.

	Before Weighting (Raw)				After Overlap Weighting (Weighted)			
Variable	K-Wire (*n* = 50)	Screw/ Plate (*n* = 12)	*p*-Value	SMD	K-Wire (wN = 8.42)	Screw/ Plate (wN = 8.42)	*p*-Value	SMD
Post-traumatic OA change, *n* (%)	20 (40.0%)	7 (58.3%)	0.409	0.373	14.8 (29.6%) ^‡^	6.9 (57.7%) ^‡^	0.091	0
C1-M2 gap (mm) ^†^	2.06 (0.86)	2.67 (0.98)	0.034	0.667	1.919 (0.115) ^†^	2.536 (0.279) ^†^	0.046 *	0
Mean diff. (95% CI)	-	-		-	0.617 (0.013–1.220)	-		-

Values are presented as mean (SD), number (%), weighted mean (standard error), or weighted proportion, as appropriate. ^†^ Values are weighted means (standard errors) adjusted by overlap weighting. ^‡^ Values are weighted proportions. * *p* < 0.05. CI, confidence interval; OA, osteoarthritis; SMD, standardized mean difference.

## Data Availability

The data presented in this study are available from the corresponding author upon request.

## Abbreviations

The following abbreviations are used in this manuscript:

ATO	Average treatment effect in the overlap population
BMI	Body mass index
C1	Medial cuneiform
C2	Intermediate cuneiform
C3	Lateral cuneiform
C1-M2	Medial cuneiform–second metatarsal
CI	Confidence interval
CT	Computed tomography
Cu	Cuboid
IQR	Interquartile range
K-wire	Kirschner wire
M1	First metatarsal
M2	Second metatarsal
M3	Third metatarsal
OA	Osteoarthritis
PS	Propensity score
SD	Standard deviation
SE	Standard error
SMD	Standardized mean difference
wN	Weighted sample size

## References

[1] Nunley J.A., Vertullo C.J. (2002). Classification, investigation, and management of midfoot sprains: Lisfranc injuries in the athlete. Am. J. Sports Med..

[2] Kuo R.S., Tejwani N.C., Digiovanni C.W., Holt S.K., Benirschke S.K., Hansen S.T., Sangeorzan B.J. (2000). Outcome after open reduction and internal fixation of Lisfranc joint injuries. J. Bone Jt. Surg. Am..

[3] Vuori J.P., Aro H.T. (1993). Lisfranc joint injuries: Trauma mechanisms and associated injuries. J. Trauma.

[4] Myerson M.S. (1999). The diagnosis and treatment of injury to the tarsometatarsal joint complex. J. Bone Jt. Surg. Br..

[5] Moracia-Ochagavía I., Rodríguez-Merchán E.C. (2019). Lisfranc fracture-dislocations: Current management. EFORT Open Rev..

[6] Weatherford B.M., Anderson J.G., Bohay D.R. (2017). Management of tarsometatarsal joint injuries. J. Am. Acad. Orthop. Surg..

[7] Mulier T., Reynders P., Dereymaeker G., Broos P. (2002). Severe Lisfrancs injuries: Primary arthrodesis or ORIF?. Foot Ankle Int..

[8] Arntz C.T., Veith R.G., Hansen S.T. (1988). Fractures and fracture-dislocations of the tarsometatarsal joint. J. Bone Jt. Surg. Am..

[9] Hardcastle P.H., Reschauer R., Kutscha-Lissberg E., Schoffmann W. (1982). Injuries to the tarsometatarsal joint. Incidence, classification and treatment. J. Bone Jt. Surg. Br..

[10] Myerson M.S., Fisher R.T., Burgess A.R., Kenzora J.E. (1986). Fracture dislocations of the tarsometatarsal joints: End results correlated with pathology and treatment. Foot Ankle.

[11] Sheibani-Rad S., Coetzee J.C., Giveans M.R., DiGiovanni C. (2012). Arthrodesis versus ORIF for Lisfranc fractures. Orthopedics.

[12] Poutoglidou F., van Groningen B., McMenemy L., Elliot R., Marsland D. (2024). Acute Lisfranc injury management. Bone Jt. J..

[13] O’Connor K.P., Tackett L.B., Riehl J.T. (2024). Primary arthrodesis versus open reduction internal fixation for acute Lisfranc injuries: A systematic review and meta-analysis. Arch. Orthop. Trauma Surg..

[14] Cochran G., Renninger C., Tompane T., Bellamy J., Kuhn K. (2017). Primary arthrodesis versus open reduction and internal fixation for low-energy Lisfranc injuries in a young athletic population. Foot Ankle Int..

[15] Ly T.V., Coetzee J.C. (2006). Treatment of primarily ligamentous Lisfranc joint injuries: Primary arthrodesis compared with open reduction and internal fixation. A prospective, randomized study. J. Bone Jt. Surg. Am..

[16] Hu S.J., Chang S.M., Li X.H., Yu G.R. (2014). Outcome comparison of Lisfranc injuries treated through dorsal plate fixation versus screw fixation. Acta Ortop. Bras..

[17] Stavrakakis I.M., Magarakis G.E., Christoforakis Z. (2019). Percutaneous fixation of Lisfranc joint injuries: A systematic review of the literature. Acta Orthop. Traumatol. Turc..

[18] Delman C., Patel M., Campbell M., Kreulen C., Giza E. (2019). Flexible fixation technique for Lisfranc injuries. Foot Ankle Int..

[19] Nery C., Baumfeld D., Baumfeld T., Prado M., Giza E., Wagner P., Wagner E. (2020). Comparison of suture-augmented ligamentplasty to transarticular screws in a Lisfranc cadaveric model. Foot Ankle Int..

[20] Herscovici D., Scaduto J.M. (2018). Acute management of high-energy lisfranc injuries: A simple approach. Injury.

[21] Qu W., Ni S., Wang Z., Zhao Y., Zhang S., Cheng Y., Liu T., Yu M., Wang D. (2016). Severe open Lisfranc injuries: One-stage operation through internal fixation associated with vacuum sealing drainage. J. Orthop. Surg. Res..

[22] Lee C.A., Birkedal J.P., Dickerson E.A., Vieta P.A., Webb L.X., Teasdall R.D. (2004). Stabilization of Lisfranc joint injuries: A biomechanical study. Foot Ankle Int..

[23] de Palma L., Santucci A., Sabetta S.P., Rapali S. (1997). Anatomy of the Lisfranc joint complex. Foot Ankle Int..

[24] Wiley J.J. (1971). The mechanism of tarso-metatarsal joint injuries. J. Bone Jt. Surg. Br..

[25] Seo D.K., Lee H.S., Lee K.W., Lee S.K., Kim S.B. (2017). Nonweightbearing radiographs in patients with a subtle Lisfranc injury. Foot Ankle Int..

[26] Kellgren J.H., Lawrence J.S. (1957). Radiological assessment of rheumatoid arthritis. Ann. Rheum. Dis..

[27] Li F., Morgan K.L., Zaslavsky A.M. (2017). Balancing covariates via propensity score weighting. J. Am. Stat. Assoc..

[28] Li F., Thomas L.E., Li F. (2019). Addressing extreme propensity scores via the overlap weights. Am. J. Epidemiol..

[29] Austin P.C., Stuart E.A. (2015). Moving towards best practice when using inverse probability of treatment weighting (IPTW) using the propensity score to estimate causal treatment effects in observational studies. Stat. Med..

[30] Adachi A., Takegami Y., Nakashima H., Mishima K., Kobayashi K., Imagama S. (2025). Comparative clinical outcomes of K-wire fixation versus screw fixation in Lisfranc joint injuries: A multicenter (TRON group) retrospective study. J. Foot Ankle Surg..

[31] Dubois-Ferrière V., Lübbeke A., Chowdhary A., Stern R., Dominguez D., Assal M. (2016). Clinical outcomes and development of symptomatic osteoarthritis 2 to 24 years after surgical treatment of tarsometatarsal joint complex injuries. J. Bone Jt. Surg. Am..

